# Tobacco control is integral to global COVID-19 response: an opportunity to accelerate toward SDGs

**DOI:** 10.1093/eurpub/ckac131.065

**Published:** 2022-10-25

**Authors:** DTH Chen

**Affiliations:** Imperial College London, London, UK

## Abstract

**Issue:**

Tobacco use intersects with the COVID-19 pandemic not only in terms of health consequences, but also on public health systems, economies, and the environment.

**Description of the problem:**

The global tobacco supply contributes significantly to environmental pollution of the natural ecosystems. The damage is compounded by tobacco consumption and its resultant waste, which leaves a significant carbon footprint on the environment, undermining the planet’s ecological stability and intensifying climate change. Furthermore, tobacco use exacerbates inequalities and adds burdens to COVID-19-related mortality, which are major challenges to recovery from the COVID-19 pandemic.

**Results:**

he pandemic has provided a chance to combat tobacco use and accelerate efforts to alleviate these challenges and accelerate progress toward Sustainable Development Goals (SDGs). The MPOWER measures from the World Health Organization Framework Convention on Tobacco Control (WHO FCTC) can play an integral part to boost sustainable and equitable COVID-19 recovery — Monitor tobacco use (article 20); Protection from tobacco smoke (article 8); Offer help for tobacco cessation (article 14); Warn about the dangers of tobacco (article 11); Enforcing bans on tobacco advertising, promotion, and sponsorship (TAPS) (article13); and Raise tobacco taxes (article 6).

**Lessons:**

To accelerate recovery, it is critical to call for actions for governments and policy-makers to strengthen synergies and policy actions to emphasise tobacco control across equity, public health, climate actions, and counteract against the tobacco industry during and beyond COVID-19 as global authorities pledge to achieve the SDGs.

**Key messages:**

• Global authorities must create better synergies on policies with a prime focus on reinforcing tobacco control to recover from the pandemic.

• The WHO FCTC MPOWER measures can play an integral part in COVID-19 recovery to fight tobacco use and accelerate progress toward SDGs.

